# Preliminary Screening for the Anthelmintic Activity of *Millettia zechiana* Harms and Its Modifying Effect on Albendazole

**DOI:** 10.1155/2024/5513489

**Published:** 2024-05-02

**Authors:** Michael Asah Asiamah, Theresa Appiah Agana, Yaw Duah Boakye, Christian Agyare, Francis Adu

**Affiliations:** Department of Pharmaceutics, Faculty of Pharmacy and Pharmaceutical Sciences, Kwame Nkrumah University of Science and Technology, Kumasi, Ghana

## Abstract

Helminthic infections affect a greater proportion of the world's population. This study determined the anthelmintic activity of *Millettia zechiana* and its modifying effect on albendazole. Powdered leaves of *M. zechiana* were successively extracted with petroleum ether, ethyl acetate, and ethanol. The anthelmintic potential of the *M. zechiana* leaf extracts and the modifying effects of the extracts on albendazole were determined on *Pheretima posthuma*. Phytochemical and gas chromatography-mass spectroscopy (GC-MS) analyses were performed to determine the chemical composition of each extract. The plant extracts of *M. zechiana* had few or all phytoconstituents such as tannins, saponins, flavonoids, glycosides, terpenoids, phytosterols, and alkaloids present. The IC_50_ obtained for albendazole, petroleum ether, ethyl acetate, and ethanol extracts for paralysis time were 0.936, 1.722, 1.283, and 1.348 mg/mL, respectively. The IC_50_ obtained for albendazole and the ethanol extract for death time were 4.638 and 4.988 mg/mL. The ethanol extract at 10 and 5 mg/mL caused death in the worms after 152.5 ± 8.66 minutes and 304.8 ± 7.27 minutes of exposure, respectively. Ethanol, ethyl acetate, and petroleum ether extracts of *M. zechiana* significantly modified the activity of albendazole at concentrations of 2.5 and 1.25 mg/mL (*P* < 0.0001). The ethanol extract which exhibited the best anthelminthic activity was fractionated through column chromatography, and five (5) fractions were obtained. Fractions 1, 2, 4, and 5 had the best paralytic activities against the worms. Fractions 1 and 2 demonstrated better helminthicidal activity than albendazole, which had an IC_50_ of 3.915. The GC-MS analysis of the ethanol, ethyl acetate, and petroleum ether extracts showed the presence of 10, 10, and 37 compounds, respectively, with 9-octadecenamide, (Z)-, n-hexadecanoic acid, oleic acid, and some aromatic compounds being the most predominant. The results obtained indicate that *M. zechiana* leaf extract possesses anthelmintic activity.

## 1. Introduction

Helminth infections impact a greater proportion of the world's population, and they are known to be the commonest parasitic infections in man, livestock, and wildlife [1]. Soil-transmitted helminth infections are pervasive in many developing countries and represent the most widespread infectious agents [2]. The most prevalent helminth infections are caused by soil-transmitted helminths (STHs), and these organisms reside within the intestines of both humans and livestock animals [3]. The infections are prevalent in tropical and subtropical areas such as China, Africa, East Asia, and the Americas. The infection can be linked to a lack of personal hygiene, poor sanitary practices, and an inadequate supply of water which are major challenges in most developing countries [2].

Helminth infections are usually transmitted through ingestion of the eggs of the worm present in contaminated food or water. Helminths depend on the host they infect for nourishment and protection while causing harm to the living host. In humans, worm infestations usually cause childhood malnutrition anaemia and gastrointestinal infections, which usually result in high morbidity in children [4]. Helminth infections are not easily detected since the associated symptoms are not easily recognised [5]. Access to conventional therapy for helminth infections in the developing world is a great challenge [6]. Therefore, more convenient alternative sources of anthelmintic agent need to be investigated.

Plants have served as a major source of secondary metabolites with several reported pharmacological activities. They have long been used for different applications such as traditional medicines, food additives, flavourings, and preservatives [7, 8]. Demand for medicinal plants in recent times has increased significantly because they have been proven to have bioactive compounds and fewer side effects [9].

*Millettia* are members of the Fabaceae family, which has over 200 species found throughout the tropical and subtropical areas of the world such as Asia, Africa, and the Caribbean. Plants in this genus are commonly used as a remedy for a variety of illnesses, including helminth infections, wounds, skin disorders, sores, snake bites, muscle aches, boils, and gynaecological diseases [10]. In Ghana, *Millettia zechiana* Harms is used in the management of new and old wounds [11].

The commonly used drugs used in treating soil-transmitted helminthic infections include albendazole, mebendazole, levamisole, and pyrantel pamoate [12, 13]. However, reports indicate that the efficacy of two of these drugs, albendazole and mebendazole, has reduced over time and therefore the urgent need to search for alternatives [14]. It is necessary to identify natural biologically active compounds that possess anthelminthic properties since increased resistance to current treatment regimens for helminth infection is on the rise [14]. This study, therefore, examined the anthelmintic potential of *M. zechiana* leaves and assessed the modifying effect the plant could have on one of the commonly used drugs, albendazole.

## 2. Materials and Methods

### 2.1. Collection of Specimens, Identification, and Authentication

The plant sample was obtained from forests and farms in Ejisu-Boankra (latitude 6° 42′ 12^″^ N, longitude 1° 24′ 52^″^ W, elevation 234 m) in the Ashanti Region of Ghana between September and November 2021. Fresh *M. zechiana* leaf samples were collected before sunrise. The leaf was identified and verified by Clifford Asare, a botanist from the Department of Herbal Medicine at the Faculty of Pharmacy and Pharmaceutical Sciences at KNUST in Kumasi, Ghana. A voucher sample (KNUST/HMI/2022 L017) was also reserved for reference purposes at the same department.

### 2.2. Chemicals and Reagents

All the chemicals and reagents used in the study were purchased in the highest quality available from Sigma-Aldrich, London, UK, unless otherwise stated.

### 2.3. Preparation of *M. zechiana* Extracts

To prepare *M. zechiana* leaves for analysis, they were first washed and left to air-dry at room temperature for five (5) to seven (7) days. Once completely dried, the leaves were finely ground into a powder using a milling machine (Christy and Norris, Chelmsford, UK). The resulting powder was then carefully kept in a desiccator for future analysis.

The process described by Sharma [15] was used to successively extract *M. zechiana* leaves with solvents of increasing polarity (petroleum ether, ethyl acetate, and ethanol). The plant material was completely dried for two (2) days after each solvent extraction before extracting with successive solvents. Powdered samples (100 g) were weighed into conical flasks containing 1000 mL of solvent and stoppered with a cork to prevent the solvent from evaporating. The samples were kept on a rotary shaker at 200 rpm for 48 hours followed by sieving the extracts using a sterile Whatman no. 1 filter paper (Sigma-Aldrich, London, UK). Using a rotary evaporator (Rotavapor BÜCHI R-200 with heating bath B-490, Büchi, Konstanz) at 40°C under vacuum, the solvents were removed, and the dried extracts were kept in sterile bottles and refrigerated.

### 2.4. Phytochemical Screening of *M. zechiana* Extracts

The plant powder, as well as ethanol, ethyl acetate, and petroleum ether extracts, was screened for their phytochemical constituents (alkaloids, saponins, cardiac glycosides, flavonoids, phenols, triterpenoids, and tannins). The phytoconstituents were identified by characteristic colour change using standard procedures by Sikandar et al. [16], performing each experiment in triplicate.

### 2.5. Collection of Worms

Adult worms (*Pheretima posthuma*) were dug out from the wet soil from the Wiwi River which runs adjacent to the Department of Theoretical and Applied Biology at KNUST, situated at a latitude of 6° 35 N and a longitude of 1° 35 W. The type of earthworm was validated at the Zoology Division, Department of Theoretical and Applied Biology, KNUST, and the worms were rinsed with normal saline to eliminate the soil and debris.

### 2.6. Anthelmintic Activity of the Plant Extract

The approach used by Osei Akoto et al. [17] was used to uncover the antiparasitic potential of the plant sample. The study involved the preparation of four different concentrations of the plant extract (10, 5, 2.5, and 1.25 mg/mL) for *in vitro* assessment of its anthelmintic action. The experiment was further augmented by using albendazole (Fisher Scientific, Schwerte, Germany) (20 mg/mL) as a positive control and distilled water as a negative control. Three adult worms of *P. posthuma* roughly the same length (10.5 cm) were placed into different Petri dishes having 20 mL of each of the ethanol, ethyl acetate, and petroleum ether extracts of *M. zechiana*. The worms were observed for paralysis and/or death for 480 minutes. The time of paralysis was precisely established when not a single twitch could be observed unless the worms were subjected to intense jostling and death was verified when the worms were not moving even upon rigorous agitation. The death of the worms was confirmed when no movement was detected even after being dipped into warm water at 45°C. The entire experiment was repeated three times.

### 2.7. Anthelmintic Modulatory Activity of the Plant Extracts

The approach utilized by Wahab Obeng et al. [18] was used to assess the anthelmintic modifying properties of extracts derived from *M. zechiana*. Upon testing various concentrations of these extracts, it was found that concentrations of 1.25 mg/mL did not induce paralysis or death in the worms, and this concentration was therefore designated as the subinhibitory concentration for the plant extracts. Albendazole (20 mg/mL) was added to a subinhibitory concentration of each plant extract to obtain concentrations of 10 mg/mL. This concentration was serially diluted to 5, 2.5, and 1.25 mg/mL by combining it with the subinhibitory concentration of the *M. zechiana* extract. For each test solution, three worms that were roughly 10.5 cm in size were placed in individual Petri dishes having 20 mL of the solution. The worms were observed for paralysis and/or death for 480 minutes. The death of the worms was recorded when they failed to respond to vigorous shaking, and their death was confirmed by placing them in warm water at 45°C and checked for movement. The above experiment was performed in triplicate.

### 2.8. Gas Chromatography-Mass Spectrometry Analysis of Extracts

The method described by Wahab Obeng et al. [18] was used to determine the compounds present in the various extracts of *M. zechiana*. The extracts of *M. zechiana* were analyzed using a PerkinElmer GC Clarus 580 Gas Chromatograph attached to a PerkinElmer Clarus SQ 8S Mass Spectrometer. One (1) milligram of dried *M. zechiana* extracts was dissolved in their respective solvents used in their extraction and then injected into the inlet chamber of the gas chromatograph (GC). The chromatograph was equipped with a ZB-5HTMS column (30 × 0.25 *μ*m ID × 0.25 *μ*m DF). The mobile phase used was helium, and the steady flow rate was fixed at 1 mL/min. The temperatures for the injectors and the oven were set at 250°C and 100°C (for two (2) minutes), respectively. The temperature of the oven was gradually elevated to 280°C, and the temperature was maintained for ten (10) minutes. Mass spectra were taken at 70 eV, 0.5 seconds of scan interval and fragments from 40 to 550 Da. The entire GC/MS run lasted a total of 38 minutes, during which the data was thoroughly evaluated by referencing the extensive database of the National Institute of Standards and Technology (NIST), which has a collection of over 62,000 spectrum patterns.

### 2.9. Column Chromatography

Singh et al. [19] described a method used for fractionating crude plant extracts and that was used to fractionate the ethanol extract of *M. zechiana*, as it displayed the strongest anthelmintic activity. This method involved determining the most suitable solvent, which was found to be a combination of one-part ethyl acetate with four-part petroleum ether, using thin-layer chromatography. Twenty grams (20 g) of the dry ethanol extract was dissolved in ethanol and mixed with 60 g of silica (Fisher Scientific, Schwerte, Germany); then, the resulting slurry was dried to remove the solvent. Silica with a mesh size of 70 to 230 was used in the process. The ethanol extract containing silica was transferred into a cylindrical glass tube of 90 × 5 cm with a plugged bottom and a cotton plug placed at the surface to prevent solvent disturbance. A litre of petroleum ether was run through the column to eliminate air bubbles. To extract the desired compounds, the solvent system of one-part ethyl acetate to four-part petroleum ether was used, with increasing polarity from 1 : 4 to 1.5 : 3.5, 2 : 3, 3 : 2, and 3.5 : 1.5 (ethyl acetate : petroleum ether) resulting in 18 fractions. The fractions were combined using thin-layer chromatography to obtain five final fractions, which were then assayed for their anthelmintic activity.

## 3. Statistical Analysis

GraphPad Prism version 9.0 for Windows (GraphPad Software Inc., San Diego, CA, USA) was utilized to analyze the data obtained from the study at a significance level of *P* < 0.05. The EC_50_ of *M. zechiana* extracts, fractions, and albendazole was determined, and 100% paralysis or death was determined as 0 minutes, and 0% paralysis or death was determined as 480 minutes. The results of the anthelmintic modifying activity of albendazole by the plant extracts were examined using one-way ANOVA, followed by Dunnett's post hoc test to determine how the extract's activity varies from the albendazole drug used as a positive control.

## 4. Results

### 4.1. Percentage Yield of *M. zechiana* Extracts

The yield of phytochemicals obtained from each solvent extract showed that the ethanol and ethyl acetate extracts had the highest yields (4.71 and 4.14, respectively), compared to the petroleum ether extract which yielded 2.78% (Table 1).

### 4.2. Phytochemical Screening

The phytochemical analysis showed that the ethanol extract contains tannins, saponins, flavonoids, glycosides, terpenoids, and phytosterols, which were also present in the powdered plant. However, the ethyl acetate and petroleum ether extracts were found to contain only alkaloids, phytosterols, and terpenoids (Table 2).

### 4.3. Anthelmintic Activity of *M. zechiana* Extracts

The ethanol extract at 10 mg/mL caused paralysis at 30.25 ± 9.179 minutes from the time of exposure of the worms to the extract. However, at 5 mg/mL, paralysis occurred within 61.5 ± 8.66 minutes. At 2.5 mg/mL, paralysis of the worms occurred after 146.0 ± 9.381 minutes. The lowest concentration of the ethanol extract (1.25 mg/mL) caused no paralysis in the adult worms after 480 minutes, which was the maximum time of exposure. The ethyl acetate extract at 2.5 mg/mL caused paralysis in 114.8 ± 11.0 minutes. When exposed to 5 and 10 mg/mL of the extract, the paralysis time decreased to 79.75 ± 12.37 and 56.25 ± 6.24 minutes, respectively. However, the extract failed to induce any paralysis at 1.25 mg/mL. The times taken to cause paralysis in the worms when exposed to the petroleum ether extract at 2.5, 5, and 10 mg/mL were 162.0 ± 7.26, 57.75 ± 3.59, and 45.25 ± 8.38 minutes, respectively. There was no paralysis at 1.25 mg/mL of the petroleum ether extract (Table 3).

The ethyl acetate and petroleum ether extracts had no lethal effect on the worms after 480 minutes. However, only the ethanol extract at 5 and 10 mg/mL was lethal to the worms after 304.8 ± 7.27 and 152.5 ± 8.66 minutes of exposure, respectively (Table 3).

The percentage (%) paralysis and death time values were used together with the log concentration values (Table 4) to plot a graph, and the IC_50_ of the various extracts were obtained (Table 5). The IC_50_ obtained for albendazole, petroleum ether, ethyl acetate, and ethanol extracts for paralysis time were 0.936, 1.722, 1.283, and 1.348 mg/mL, respectively. The IC_50_ obtained for albendazole and the ethanol extract for death time were 4.638 and 4.988 mg/mL. However, the petroleum and ethyl acetate extracts had no IC_50_ values for the death time since they did not exhibit any lethal effects on the worms (Table 5).

### 4.4. Influence of *M. zechiana* Extracts on the Anthelmintic Activity of Albendazole

Albendazole alone at 1.25, 2.5, 5, and 10 mg/mL caused paralysis at 129.7 ± 11.24, 66.75 ± 3.30, 21.67 ± 0.58, and 8.00 ± 1.73 minutes, respectively, but no death of adult worms at 2.5 and 1.25 mg/mL after 480 minutes of exposure. However, at 5 and 10 mg/mL, albendazole caused the death of adult worms at 39.67 ± 5.03 and 18.0 ± 3.61 minutes, respectively (Table 6).

The time of paralysis at 1.25, 2.5, 5.0, and 10 mg/mL of ethanol extract combined with albendazole was 139.33 ± 7.57, 19.33 ± 2.08, 11.0 ± 1.73, and 5.33 ± 1.528 minutes, respectively (Table 6). The lethal effect of ethanol extract combined with albendazole at 1.25, 2.5, 5.0, and 10 mg/mL was recorded at 321.7 ± 2.08, 243.0 ± 5.29, 38.00 ± 6.56, and 10.0 ± 2.00 minutes, respectively (Table 6). The time of paralysis at 1.25, 2.5, 5.0, and 10 mg/mL of the ethyl acetate extract combined with albendazole was 30.33 ± 4.62, 13.33 ± 1.53, 9.33 ± 2.52, and 2.67 ± 0.58 minutes, respectively (Table 6). The lethal effects of ethyl acetate extract combined with albendazole at 1.25, 2.5, 5.0, and 10 mg/mL caused the death of worms after 114.33.0 ± 11.02, 69.33 ± 5.69, 24.0 ± 6.56, and 9.33 ± 3.06 minutes, respectively (Table 6). The time of paralysis at 1.25, 2.5, 5.0, and 10 mg/mL of petroleum ether extract combined with albendazole was 94.33 ± 4.45, 67.00 ± 4.00, 21.67 ± 0.58, and 8.03 ± 1.62 minutes, respectively (Table 6). The lethal effect of petroleum ether extract combined with albendazole at 5.0 and 10 mg/mL was recorded at 146.80 + 15.27 and 19.4 + 2.07 minutes, respectively. However, at 2.5 and 1.25, combined petroleum ether extract and albendazole could not cause the death of the worms (Table 6).

### 4.5. Column Chromatography Separation of *M. zechiana* Ethanol Extract

**Figure 1:**
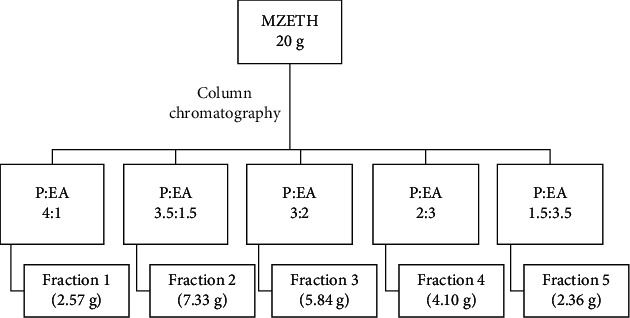
A schematic diagram for the separation of ethanol extract of *M. zechiana*. MZETH: *M. zechiana* ethanol extract; P: petroleum ether; EA: ethyl acetate.

The ethanol extract of *M. zechiana* exhibited the highest anthelmintic activity compared to the ethyl acetate and petroleum ether extracts (Table 3). Further analyses were carried out to determine which fractions contributed to the high anthelmintic activity reported for the ethanol extract. The ethanol extract was therefore fractionated through column chromatography to obtain 5 fractions (Figure 1).

### 4.6. Anthelmintic Activities of the Fractions of *M. zechiana* Ethanol Extract

The fractions obtained from the ethanol extract of *M. zechiana* were tested *in vitro* against *P. posthuma* worms. Fraction one (1) at 10, 5, 2.5, and 1.25 mg/mL caused paralysis in 19 ± 5.29, 48 ± 5.57, 64.67 ± 5.69, and 86.67 ± 1.53 minutes, respectively, from the time of exposure of the worms to the fraction (Table 7). The helminthicidal activities at 10, 5, 2.5, and 1.25 mg/mL of fraction 1 were recorded within 158.67 ± 6.03, 188.67 ± 3.21, 234.00 ± 10.54, and 275.33 ± 9.07 minutes, respectively. The paralysis and helminthicidal activities of the other fractions were also recorded (Table 7). Albendazole was used as the positive control, and the time of paralysis at 10 mg/mL was 9.52 ± 2.87 minutes, followed by death after 39.67 ± 5.033 minutes of exposure. Albendazole at 5, 2.5, and 1.25 mg/mL caused paralysis in the worms at 22.5 ± 3.42, 66.75 ± 3.30, and 138.5 ± 19.91 minutes, respectively, from the time of exposure (Table 7). At 10 mg/mL of albendazole, all worms died in 18.00 ± 3.61 minutes. At a 5 mg/mL concentration of albendazole, the helminthicidal activity was recorded at 39.67 ± 5.03 minutes. The lowest concentrations of albendazole at 2.5 and 1.25 mg/mL were not lethal to the worms (Table 7).

The percentage (%) paralysis and death time values of the ethanol fractions were used together with the log concentration values (Table 8) to plot a graph, and the IC_50_ of the ethanol fractions were obtained (Table 9). The IC_50_ values for the various fractions of ethanol extract of *M. zechiana* were compared to determine which of the fractions was very efficacious in causing paralysis in the worms. Fractions 1, 2, 3, 4, and 5 had IC_50_ values of 0.331, 0.478, 0.495, 0.359, and 0.437 mg/mL, respectively, for paralysis of worms (Table 9). Fractions 1, 2, 4, and 5 had the best paralytic activities against the worms since lower IC_50_ values were reported when compared to albendazole which had an IC_50_ value of 0.469. The IC_50_ values obtained for fractions 1, 2, 3, and 5 were 2.663, 2.579, 19.91, and 13.01 mg/mL, respectively, for the lethal effect on worms. Fractions 1 and 2 demonstrated the best helminthicidal activity than albendazole, which had an IC_50_ of 3.915 (Table 9).

### 4.7. Gas Chromatography-Mass Spectrometry Analysis (GC-MS)

**Figure 2:**
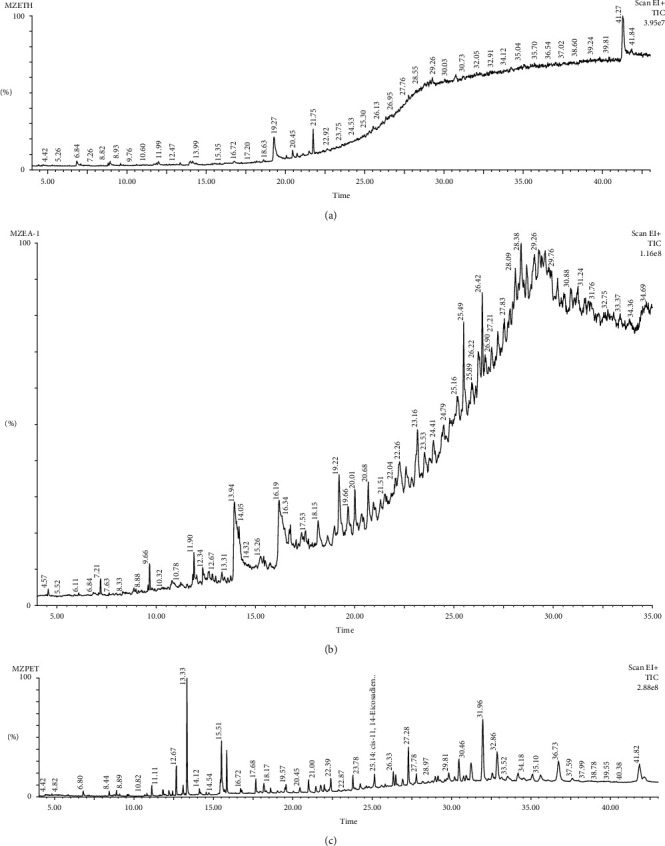
GC-MS chromatogram of the (a) ethanol, (b) ethyl acetate, and (c) petroleum ether extracts of *M. zechiana*.

GC-MS analysis was used since it can detect and identify very small quantities of analytes in complex mixtures with high accuracy and specificity. Ten (10) peaks were detected in the crude extracts of ethyl acetate and ethanol using gas chromatography-mass spectrometry analysis which showed the presence of ten compounds with their retention times ranging between 10 and 21 minutes for ethyl acetate extract and 6 to 22 minutes for the ethanol extract (Table 10 and Figure 2(b) and Table 11 and Figure 2(a), respectively). The petroleum ether extract of *M. zechiana* presented 37 peaks indicating the presence of 37 different compounds which had retention times ranging from 11.113 to 42.136 minutes (Table 12 and Figure 2(c)). The most predominant compound detected in the ethanol extract was 9-octadecenamide (Z)- (Table 11). The ethyl acetate extract had n-hexadecanoic acid and oleic acid as the most predominant (Table 10). The petroleum ether extract of *M. zechiana* had most of its components being aromatic compounds (Table 12). The most abundant compounds were cholest-4-en-3-one (8.332%), hexadecenoic acid-methyl ester (6.351%), stigmasta-4,24(28)-dien-3-one, (24E)- (5.33%), 11-octadecenoic acid-methyl ester (5.2%), and Ergosta-4,22-dien-3-one (4.884%).

## 5. Discussion

Powdered *M. zechiana* leaves were successively extracted using petroleum ether, ethyl acetate, and ethanol. The successive extraction method was used since it offers high extraction efficiency and allows for the extraction of compounds in a selective manner [20]. The highest extraction yields were obtained from ethanol and ethyl acetate extracts (4.71 and 4.14, respectively), compared to the petroleum ether extract which yielded 2.78%. The yield of the ethanol extract of *M. zechiana* was the highest among the different solvents used. As the polarity of the solvent decreased, the yield of the leaf extract also decreased (Table 1), indicating that the extract of *M. zechiana* contains many polar constituents. Similar results have been found in studies by Bahiru et al. [21] and Mouafo et al. [22] on other species of *Millettia*, where polar solvents resulted in higher extraction yields compared to nonpolar solvents like ethyl acetate and petroleum ether. This suggests that the bioactive components in the *M. zechiana* leaves are polar.

The preliminary anthelmintic activity of various solvent extracts of *M. zechiana* was tested *in vitro* against *P. posthuma* worms since they anatomically and physiologically resemble the human intestinal roundworm parasites [23–25]. To determine the efficacy of the different solvent extracts as anthelmintic, their activity was evaluated at 1.25, 2.5, 5, and 10 mg/mL. The effect of the extract on producing paralysis and death in *P. posthuma* was dependent on the dose and the solvent extract used. The observed variations in the anthelmintic activity of the different *M. zechiana* extracts may be ascribed to differences in their phytochemical compositions (Table 2).

The IC_50_ values for the various solvent extracts of *M. zechiana* were compared to determine which of the extracts was very efficacious in causing paralysis in the worms. The low IC_50_ of the ethanol and ethyl acetate extracts compared to the other solvent extracts (IC_50_ values of 1.348, 1.283, and 1.722 mg/mL for ethanol, ethyl acetate, and petroleum ether extracts, respectively) indicates that they had the best paralytic efficacy against the worms. Again, only the ethanol extract could cause the death of worms (Table 3). This might be due to the presence of phytochemicals such as tannins, saponins, phenolics, and terpenoids that have been reported by Tagoe et al. [26] and Wahab Obeng et al. [27] to possess anthelmintic activity. The bioactive components in the ethanol extract could be working in synergy or individually to cause paralysis or death in the worms [28]. This confirms the report by Koné et al. [29] that plants belonging to the family Fabaceae have potential anthelmintic properties, although no work has been done to determine the anthelmintic activity of *M. zechiana*. However, albendazole had an IC_50_ of 0.936 mg/mL for paralysis of worms and an IC_50_ of 4.638 mg/mL for death of worms, which was lower when compared to all solvent extracts of *M. zechiana*.

Plants are known to be abundant in phytoconstituents and, when combined with some of the anthelmintic drugs, may increase their efficacy and override the resistance mechanism of parasites [30]. The ability of *M. zechiana* to modify anthelmintic drugs was assessed using subinhibitory concentrations of ethanol, ethyl acetate, and petroleum ether extracts, each at 1.25 mg/mL.

**Figure 3:**
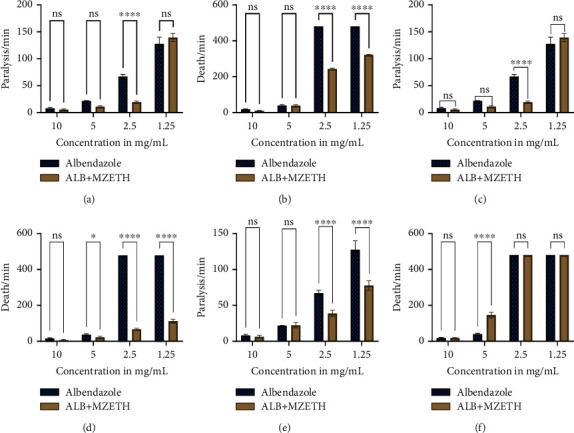
Effect of ethanol, ethyl acetate and petroleum ether extract of *M. zechiana* on albendazole. (a, c, e) Paralysis times for ethanol, ethyl acetate, and petroleum ether extracts, respectively. (b, d, f) Death times for ethanol, ethyl acetate, and petroleum ether extracts, respectively. ^∗∗∗∗^*P* < 0.0001; ns: no significant difference (*P* > 0.05).

The mean time of paralysis of the adult worms for ethanol extract combined with albendazole and albendazole only was not significant (*P* > 0.05) (Figure 3(a)). The times of death of the worms at 10 and 5 mg/mL of albendazole only and albendazole combined with ethanol extract were not significantly different, although a much lesser time was taken to cause death in the albendazole combined with the ethanol extract (10.0 ± 2.00 minutes) compared to the albendazole only (18.0 ± 3.61 minutes) (Figure 3(b)). Combining the ethanol extract with albendazole at 2.5 and 1.25 mg/mL resulted in a substantial decrease (*P* < 0.0001) in the time required to cause death in adult worms. However, at the same concentrations, unmodified albendazole was not lethal to adult worms after 480 minutes of exposure (Figure 3(b)). The ethanol extract might have increased the absorption or bioavailability of albendazole, thereby increasing its effectiveness in causing death in worms [31].

The reduction in the paralysis time of the adult worm was not significant for modified albendazole (albendazole + ethyl acetate extract) at concentrations of 10 mg/mL and 5 mg/mL (*P* > 0.05) despite the decrease in time recorded for the modified albendazole (Figure 3(c)). However, there was a significant decrease in the time needed to cause paralysis in the adult worms at concentrations of 2.5 and 1.25 mg/mL (*P* < 0.05) (Figure 3(c)). There was no significant reduction in the time taken for modified albendazole (albendazole + ethyl acetate extract) to cause death in the worms at the concentration of 10 mg/mL (*P* > 0.05) although the time of death was reduced to 9.33 ± 3.06 minutes (Figure 3(d)). At an albendazole concentration of 5 mg/mL, the time taken to kill the matured worms reduced significantly (*P* < 0.05) to 24.0 ± 6.56 minutes when the drug was modified with ethyl acetate extract (Figure 3(d)). The time of death in the worms reduced drastically (*P* < 0.0001) for the modified albendazole (albendazole + ethyl acetate) at concentrations of 1.25 (114.33 ± 11.02 minutes) and 2.5 mg/mL (69.33 ± 5.69 minutes) when compared to albendazole only at the same concentration (Figure 3(d)). Although the exact mechanism of action of the *M. zechiana* extracts is not known, the ethanol and ethyl acetate extracts might have enhanced the activity of some phytosterols which have been reported to potentially enhance the absorption of albendazole by worms [31].

There was no significant change in the paralysis or death time in the petroleum ether extract, but an increase in the death time of the worms in the presence of a subinhibitory concentration of the petroleum extract and albendazole was observed (Figure 3(f)). Phytoconstituents present within the petroleum ether extract might have reacted with albendazole to form an inactive complex which might have caused lowered absorption or affinity to the binding site of the drug leading to loss of activity of the albendazole [23, 29].

The ethanol extract of *M. zechiana* exhibited the highest anthelmintic activity compared to the ethyl acetate and petroleum ether extracts. Further analyses were carried out to determine which fractions contributed to the high anthelmintic activity reported. The ethanol extract was fractionated through column chromatography to obtain 5 fractions (Figure 1).

The fractions obtained from the ethanol extract of *M. zechiana* were tested *in vitro* against *P. posthuma* worms. The IC_50_ values for the various fractions of ethanol extract of *M. zechiana* were compared to determine which of the fractions was very efficacious in causing paralysis in the worms. Fractions 1, 4, and 5 had the best paralytic activities against the worms since lower IC_50_ values were reported when compared to albendazole (Table 7). According to Adu et al. [32], fractionation of extracts can concentrate the bioactive components, leading to higher activity in the fractions compared to the crude ethanol extract.

GC-MS analysis was used since it can detect and identify very small quantities of analytes in complex mixtures with high accuracy and specificity. The most predominant compound detected in the ethanol extract was 9-octadecenamide (Z)- which is an alkaloid and is known to have anthelmintic and antitrypanosomal properties [18]. The ethyl acetate extract had n-hexadecanoic acid and oleic acid as the most predominant compounds (Table 10). Some biological activities such as anthelmintic, antioxidant, and hypocholesterolemic activities have been attributed to n-hexadecanoic acid [33]. Oleic acid augments the activity of antioxidants and antipolymerization agents because of their stability against oxidation [34]. The petroleum ether extract of *M. zechiana* had majority of its components being aromatic compounds (Table 12). The most abundant compounds were cholest-4-en-3-one (8.332%), hexadecenoic acid methyl ester (6.351%), stigmasta-4,24(28)-dien-3-one, (24E)- (5.33%), 11-octadecenoic acid, methyl ester (5.2%), and Ergosta-4,22-dien-3-one (4.884%). Stigmasta-4,24(28)-dien-3-one, (24E)- has been reported to exhibit potential anthelmintic and antioxidant activities [35]. Methyl ester of 11-octadecenoic acid exhibits both antioxidant and antimicrobial activities [36]. Therefore, the anthelminthic activity of *M. zechiana* could be attributed to the presence of some of these compounds. Though *P. posthuma* worms are known to resemble the human intestinal roundworms, it is reported that isolated *P. posthuma* worms from nature are usually not adaptable with artificial laboratory conditions [37], and therefore, the *in vivo* anthelminthic activity of *M. zechiana* is recommended.

## 6. Conclusion

*M. zechiana* ethanol extract had the best anthelmintic activity against the worms and could cause both paralysis and death. Combining the ethanol or ethyl acetate extracts at subinhibitory concentrations with albendazole resulted in an improvement in the activity of the drug. Column chromatography of the ethanol extract resulted in five (5) fractions. Fraction 1 had the best anthelmintic activity since paralysis and death were recorded against *P. posthuma*. Fractions 4 and 5 had good paralytic activities against *P. posthuma*. The results obtained indicate that *M. zechiana* leaf possesses anthelmintic activity. This study serves as the foundation for the isolation and characterisation of anthelminthic compounds from *M. zechiana*.

## Figures and Tables

**Table 1:** Yield of the *M. zechiana* extracts.

Type of extract	Yield in percentage (%)
Ethanol	4.71
Ethyl acetate	4.14
Petroleum ether	2.78

**Table 2:** Qualitative test for phytoconstituents of the ethanol, ethyl acetate, and petroleum ether extracts of *M. zechiana* leaves.

	Tannins	Saponins	Flavonoids	Glycosides	Terpenoids	Phytosterols	Alkaloids
Pulverized plant	+	+	+	+	+	+	+
Ethanol	+	+	+	+	+	+	+
Petroleum ether	-	-	-	-	+	+	+
Ethyl acetate	-	-	-	-	+	+	+

Presence of phytoconstituents (+); absence of phytoconstituents (-).

**Table 3:** Anthelmintic activity of albendazole and *M. zechiana* extracts.

Concentration (mg/mL)	Albendazole	Ethanol	Ethyl acetate	Petroleum ether
Paralysis				
1.25	138.5 ± 19.91	480	480	480
2.50	66.75 ± 3.30	146.0 ± 9.381	114.8 ± 11.00	162.0 ± 7.26
5.00	22.5 ± 3.42	61.5 ± 8.66	79.75 ± 12.00	57.75 ± 3.59
10.00	9.52 ± 2.87	30.25 ± 9.179	56.25 ± 6.24	45.25 ± 8.38
Death				
1.25	480	480	480	480
2.50	480	480	480	480
5.00	39.67 ± 5.03	304.8 ± 7.27	480	480
10.00	18.0 ± 3.61	152.5 ± 8.66	480	480

**Table 4:** Percentage paralysis and mortality of the *P. posthuma* by *M. zechiana* extracts.

Concentration (mg/mL)	Albendazole	Ethanol	Ethyl acetate	Petroleum ether
% paralysis				
1.25	71.15 ± 4.15	0	0	0
2.50	86.09 ± 0.69	76.09 ± 2.29	69.58 ± 1.95	69.58 ± 1.95
5.00	95.31 ± 0.71	87.19 ± 1.80	83.39 ± 2.58	83.39 ± 2.58
10.00	98.07 ± 0.60	93.69 ± 1.91	88.28 ± 1.30	88.28 ± 1.30
% death				
1.25	0	0	0	0
2.50	0	0	0	0
5.00	91.74 ± 1.05	36.04 ± 1.46	0	0
10.00	97.01 ± 0.94	69.03 ± 1.03	0	0

**Table 5:** IC_50_ values for paralysis and death time of *M. zechiana* extracts.

Extract/reference drug	Paralysis (mg/mL)	Death (mg/mL)
MZETH	1.348	4.988
MZEA	1.283	ND
MZPET	1.722	ND
Albendazole	0.936	4.638

ND: not determined; MZETH: ethanol extract; MZEA: ethyl acetate extract; MZPET: petroleum ether extract.

**Table 6:** Effect of *M. zechiana* ethanol, ethyl acetate, and petroleum ether extract on the activity of albendazole.

Concentration (mg/mL)	Albendazole	Ethanol	Ethyl acetate	Petroleum ether
Paralysis (minutes)				
1.25	129.7 ± 11.24	139.33 ± 7.57	30.33 ± 4.62	94.33 ± 64.45
2.50	66.75 ± 3.30	19.33 ± 2.08	13.33 ± 1.53	67.00 ± 4.00
5.00	21.67 ± 0.58	11.00 ± 1.73	9.33 ± 2.52	21.67 ± 0.58
10.00	8.00 ± 1.73	5.33 ± 1.528	2.67 ± 0.58	8.03 ± 1.62
Death (minutes)				
1.25	480	321.7 ± 2.08	114.33 ± 11.02	480
2.50	480	243.0 ± 5.29	69.33 ± 5.69	480
5.00	39.67 ± 5.03	38.00 ± 6.56	24.0 ± 6.56	146.80 ± 15.27
10.00	18.0 ± 3.61	10.0 ± 2.00	9.33 ± 3.06	19.4 ± 2.07

**Table 7:** Anthelmintic activity of *M. zechiana* fractions.

Concentration (mg/mL)	Albendazole	Fraction 1	Fraction 2	Fraction 3	Fraction 4	Fraction 5
Paralysis (minutes)						
1.25	138.5 ± 19.91	86.67 ± 1.53	140.67 ± 13.20	154.33 ± 11.02	77.67 ± 5.69	140.67 ± 13.20
2.50	66.75 ± 3.30	64.67 ± 5.69	56.00 ± 11.36	55.33 ± 4.04	69.67 ± 1.15	76.00 ± 5.00
5.00	22.5 ± 3.42	48.00 ± 5.57	27.67 ± 1.53	29.67 ± 4.73	45.67 ± 14.05	61.00 ± 8.19
10.00	9.52 ± 2.87	19.00 ± 5.29	13.00 ± 2.00	27.33 ± 6.11	39.67 ± 0.58	39.00 ± 7.00
Death (minutes)						
1.25	480	275.33 ± 9.07	292.33 ± 10.07	480	480	480
2.50	480	234.00 ± 10.54	223.33 ± 7.64	480	480	480
5.00	39.67 ± 5.03	188.67 ± 3.21	195.67 ± 5.69	360.00 ± 5.57	480	285.33 ± 6.51
10.00	18.0 ± 3.61	158.67 ± 6.03	127.00 ± 4.00	305.67 ± 5.03	480	265.33 ± 5.13

**Table 8:** Percentage paralysis and mortality of the *P. posthuma* by *M. zechiana* fractions.

Concentration (mg/mL)	Albendazole	Fraction 1	Fraction 2	Fraction 3	Fraction 4	Fraction 5
% paralysis						
1.25	72.99 ± 2.34	81.94 ± 0.32	70.69 ± 2.75	67.85 ± 2.295	83.82 ± 1.19	82.08 ± 1.67
2.50	86.04 ± 0.83	86.53 ± 1.19	88.33 ± 2.37	88.47 ± 0.84	85.49 ± 0.241	84.17 ± 1.04
5.00	95.42 ± 0.21	90.0 ± 1.16	94.24 ± 0.32	93.82 ± 0.99	90.49 ± 2.93	87.29 ± 1.71
10.00	98.26 ± 0.32	96.04 ± 1.10	97.29 ± 0.42	94.31 ± 1.27	91.74 ± 0.12	91.88 ± 1.46
% death						
1.25	0	42.639 ± 1.890	39.10 ± 2.097	0	0	0
2.50	0	51.25 ± 2.195	53.472 ± 1.591	0	0	0
5.00	91.74 ± 1.05	60.69 ± 0.67	59.24 ± 1.19	25.0 ± 1.16	0	40.56 ± 1.36
10.00	96.25 ± 0.75	66.94 ± 1.25	73.54 ± 0.83	36.32 ± 1.05	0	44.72 ± 1.07

**Table 9:** IC_50_ values for paralysis and death time of *M. zechiana* ethanol fractions.

Fraction/reference drug	Paralysis (mg/mL)	Death (mg/mL)
Fraction 1	0.331	2.663
Fraction 2	0.478	2.579
Fraction 3	0.495	19.91
Fraction 4	0.359	ND
Fraction 5	0.437	13.01
Albendazole	0.469	3.915

ND: not determined.

**Table 10:** GC-MS analysis of *M. zechiana* ethyl acetate extract.

No.	Retention time	Name	Molecular weight	Area	Molar mass
1	10.819	10-Acetoxy-2-hydroxy-1,2,6a,6b,9,9,12a-heptamethyl-1,3,4,5,6,6a,6b,7,8,8a,9,10,11,12,12a,12b,13,14b-octadecahydro-2H-picene-4a-carboxylic acid, methyl ester (phytosterols)	C_33_H_52_O_5_	1.729	528.8
2	11.901	Octadecane	C_18_H_38_	1.654	254.49
3	12.634	a-Amyrin (triterpene)	C_30_H_50_O	1.661	426.7
4	13.936	n-Hexadecanoic acid	C_16_H_32_O_2_	11.907	256.42
5	14.175	Eicosane	C_20_H_42_	7.194	282.5
6	16.191	Oleic acid	C_18_H_34_0_2_	15.988	282.47
7	16.760	Octadecane, 3-ethyl-5-(2-ethylbutyl)-	C_26_H_54_	3.951	366.7
8	19.217	9-Octadecenamide, (Z)- (alkaloid)	C_18_H_35_NO	6.497	281.5
9	20.005	Oleic acid, 3-hydroxypropyl ester	C_21_H_40_O_3_	3.147	340.5
10	20.684	Methyl stearate	C_19_H_38_O_2_	3.089	298.5

**Table 11:** GC-MS analysis of ethanol extract of *M. zechiana*.

No.	Retention time	Name	Molecular formula	Area	Molecular weight
1	6.840	Eugenol (phenolic acid)	C_10_H_12_O_2_	3.29	164.20
2	8.931	Phenol, 2-methoxy-4-(2-propenyl)-, acetate (phenolic acid)	C_12_H_14_O_3_	2.129	206.24
3	11.993	2,5,5,8a-Tetramethyl-4-methylene-6,7,8,8a-tetrahydro-4H,5H-chromen-4a-yl hydroperoxide	C_14_H_22_O_3_	2.712	238.32
4	13.991	n-Hexadecanoic acid	C_16_H_32_O_2_	2.654	256.42
5	14.156	Hexadecanoic acid-ethyl ester	C_18_H_36_O_2_	2.722	284.48
6	16.796	2-Hexadecanol	C_16_H_34_0	3.118	242.44
7	19.272	9-Octadecenamide, (Z)- (alkaloid)	C_18_H_35_NO	23.398	281.5
8	20.445	(2,3-Diphenylcyclopropyl)methyl phenyl sulfoxide, trans-	C_22_H_20_OS	2.771	332.5
9	21.490	Ethanol, 2-(9-Octadecenyloxy)-, (E)- (terpenoids)	C_20_H_40_O_2_	2.096	312.30
10	21.747	Diisooctyl phthalate (terpenoids)	C_24_H_38_O_4_	6.837	390.6

**Table 12:** GC-MS analysis of petroleum ether extract of *M. zechiana*.

No.	Retention time	Name	Molecular formula	Area	Molecular weight
1	11.113	Methyl tetradecanoate	C_15_H_30_O_2_	0.648	242.40
2	12.671	7-Acetyl-6-ethyl-1,1,4,4-tetramethyltetralin	C_18_H_26_O	1.915	258.40
3	13.093	9-Hexadecanoic acid, methyl ester	C_19_H_38_O_2_S_2_	0.687	362.60
4	13.331	Hexadecenoic acid, methyl ester	C_17_H_32_O_2_	6.351	268.40
5	14.120	1-Nonadecane	C_19_H_40_	0.702	268.50
6	15.513	11-Octadecenoic acid, methyl ester	C_19_H_36_O_2_	5.200	296.50
7	15.843	Methyl stearate	C_19_H_38_O_2_	2.237	298.50
8	17.677	5,8,11,14-Eicosatetraenoic acid, methyl ester, (all-Z)	C_21_H_34_O_2_	0.975	318.49
9	18.172	11,14-Eicosadienoic acid, methyl ester	C_21_H_38_O_2_	0.919	322.53
10	19.565	Tetracosane	C_24_H_50_	0.781	338.7
11	20.995	Heptacosane	C_27_H_56_	1.043	380.7
12	21.747	Diisooctyl phthalate	C_24_H_38_O_4_	0.867	390.6
13	22.389	Pentacosane	C_25_H_52_	1.273	352.7
14	23.782	Octacosane	C_28_H_58_	1.165	394.8
15	24.241	Methyl eicosa-5,8,11,14,17-pentaenoate	C_21_H_32_O_2_	0.687	316.48
16	25.139	*cis*-11,14-Eicosadienoic acid methyl ester	C_21_H_38_O_2_	1.754	322.53
17	26.331	Cholesta-4,6-dien-3-ol, (3a)-	C_27_H_44_O	1.080	384.6
18	26.918	15,17,19,21-Hexatriacontatetrayne	C_36_H_58_	0.805	490.8
19	27.284	Benzenamine,4-(1,1,3,3-tetramethylbutyl)-N-14-(1,1,3,3-tetramethylbutyl)phenyl1-	C_20_H_27_N	3.419	281.4
20	27.779	Tetratetracontane	C_44_H_90_	1.120	619.2
21	28.971	Carotene, 1,1′,2,2′-tetrahydro-1,1′-dimethoxy-	C_42_H_64_O_2_	0.772	600.49
22	29.815	Desmosterol	C_27_H_44_O	1.124	384.64
23	30.456	Cholestan-3-one, (5a)-	C_27_H_46_O	2.419	386.7
24	30.915	4,22-Cholestadien-3-one	C_27_H_42_O	0.610	382.6
25	31.960	Cholest-4-en-3-one	C_27_H_44_O	8.332	384.6
26	32.546	Cholesta-4,6-dien-3-one	C_27_H_42_O	1.211	382.6
27	32.858	Ergosta-4,22-dien-3-one	C_28_H_44_O	4.884	396.6
28	33.133	Butanoic acid, 1a, 2, 5, 5a, 6, 9, 10,10a-octahydro-5a-hydroxy-4-(hydroxymethyl)-1,1,7,9-tetramethyl-6,11-dioxo-1H-2, 8a-methanocyclopenta[a]cyclopropa[e]cyclodecen-5-yl ester, [1aR-(1aalpha,2alpha,5beta,5abeta,8aalpha,9alpha,10aalpha)]-	C_24_H_32_O_6_	0.575	41.65
29	33.518	Ergosta-4,7,22-trien-3-one	C_28_H_42_O	1.120	394.6
30	34.178	Pregn-4-ene-3,20-dione, 16-hydroxy-(16a)-	C_21_H_30_O_3_	1.694	330.5
31	34.563	Pregnan-20-one, 3-(acetyloxy)-5,6-epoxy-,3a,5a,6a)-	C_23_H_34_O_4_	0.637	374.521
32	35.095	4,22-Stigmastadiene-3-one	C_29_H_46_O	1.543	410.7
33	35.608	Cholestane-3,6,-dione, (5a)- (phytosterols)	C_27_H_44_O_2_	1.712	400.651
34	36.727	Stigmasta-4,24(28)-dien-3-one, (24E)-	C_29_H_46_O	5.333	410.355
35	37.589	Cyclopenta (d)anthracene-8,11-dione-1,2,3,3a,4,5,6,6a,7,12-decahydro-3-isopropyl-6-methylene-	C_21_H_26_O_2_	0.948	310.193
36	41.824	Benzoic acid, 3,5-dicyclohexyl-4-hydroxy-, methyl ester	C_20_H_28_O_3_	4.896	316.4
37	42.136	Propanoic acid, 3,3′-thiobis-, ditetradecyl ester	C_30_H_58_O_4_S	1.761	514.844

## Data Availability

All data used to support the findings of this study are included within the article.
